# *FAT4* expression in peripheral blood mononuclear cells is associated with prognosis and immune cell infiltration in hepatocellular carcinoma

**DOI:** 10.1038/s41598-023-42560-w

**Published:** 2023-09-21

**Authors:** Jing Li, Minling Lv, Qi Huang, Rui Hu, Xin Zhong, Xinfeng Sun, Wenxing Feng, Zhiyi Han, MengQing Ma, Wei Zhang, Xiaozhou Zhou

**Affiliations:** 1 Department of Liver Disease, Shenzhen Traditional Chinese Medicine Hospital, Shenzhen, 518033 People’s Republic of China; 2 Department of Liver Disease, The Fourth Clinical Medical School, Guangzhou University of Chinese Medicine, Shenzhen, 518033 People’s Republic of China; 3 Faculty of Chinese Medicine, Taipa, Macau University of Science and Technology, Macao, People’s Republic of China

**Keywords:** Liver cancer, Hepatocellular carcinoma

## Abstract

Peripheral blood mononuclear cell (PBMC) genes reflect the host immune status and could be suitable for evaluating the prognosis of patients with hepatocellular carcinoma (HCC), for which a reliable biomarker is unavailable and the host immune responses to cancer cells. This study aimed to investigate prognostically relevant genes in HCC PBMCs and assessed whether their expression represents tumor immune infiltration. Gene expression in PBMCs from patients with advanced or terminal HCC who had survived or died was examined. Correlations among FAT atypical cadherin 4 (*FAT4*) expression, cancer immune characteristics, and infiltrated immune cell gene marker sets were analyzed. *FAT4* expression was lower in the PBMCs of patients with advanced or terminal HCC who had died than that in patients who survived. Kaplan–Meier analysis indicated that *FAT4* downregulation was associated with a relatively poor prognosis while overexpression was positively correlated with immune cell infiltration, several immune cell markers, and immune checkpoint expression. Hsa-miR-93-5p represented the most probable upstream microRNA of *FAT4*. Thus, upregulated *FAT4* in PBMCs and HCC tissues might indicate a favorable prognosis and increased immune cell infiltration, while miRNA-93-5p could be a modulator of *FAT4* expression. Collectively, these findings suggest novel immunotherapy targets for HCC.

## Introduction

Primary liver cancer is a global health issue. Approximately 90% of primary liver cancer cases are constituted by hepatocellular carcinoma (HCC), which has a 5-year survival rate of 18%^1^. Approximately 40% of global HCC cases originate in China^1,2^. Chronic liver diseases, such as HBV- and HCV-related infections; alcoholic, metabolic, and dietary fatty liver diseases; and autoimmune or chronic cholesterol diseases and HCC exhibit a cause-and-effect relationship^3^. Although surgical interventions can be effective in the early stages of HCC, most patients are diagnosed at advanced stages and thus are often ineligible for surgery^4^. Rapid tumor progression and metastasis are the main contributors to the short overall survival and high mortality rates in patients with advanced HCC^5^. For patients unable to undergo surgery, molecular-targeted therapy and immune checkpoint inhibitors are commonly administered^4^. However, tumor-infiltrating immune cells, such as tumor-associated macrophages^6^, affect the prognosis and efficacy of chemotherapy and immunotherapy and impact tumor angiogenesis, progression, and metastasis^7,8^. Nevertheless, reliable immune-related biomarkers to evaluate prognosis and immune infiltration in advanced HCC is currently unavailable.

With regards to the immune system functioning against cancer, peripheral blood mononuclear cells (PBMCs), such as monocytes and lymphocytes, are the first line of defense^9^. Through direct interactions, cancer cells induce various phenotypic and functional changes in immune cells. Differential expression of PBMCs genes may be related to cancer immunogenicity or immune evasion^10,11^. One study found that expanded T cell receptor clones of newly infiltrating CD8 + T cells in tumors were significantly correlated with expanded peripheral clones^12^. Moreover, patients that respond well to programmed cell death protein 1/programmed cell death protein ligand 1 blockade have persistent tumor immune circulating activity in vivo, indicating that CD8 + T cells in HCC tumor tissues can be replenished by infiltrating peripheral CD8 + T cells. This in turn suggests that peripheral immune cells may reflect local immune cell invasion in the tumor microenvironment^13,14^. Therefore, the differential expression of genes in PBMCs may reflect the host’s immune status and could be used to analyze peripheral immune cells potentially suitable for evaluating the prognosis of patients with HCC and the host immune responses to cancer cells^12^.

Hence, as an approach to identify reliable biomarkers for HCC, we sought to investigate whether the expression of prognostically relevant genes, including *FAT4* (FAT atypical cadherin 4), in PBMCs and HCC tissues represent useful diagnostic or prognostic markers.

## Results

### Identification of prognostically relevant genes in PBMCs of patients with HCC

**Figure 1:**
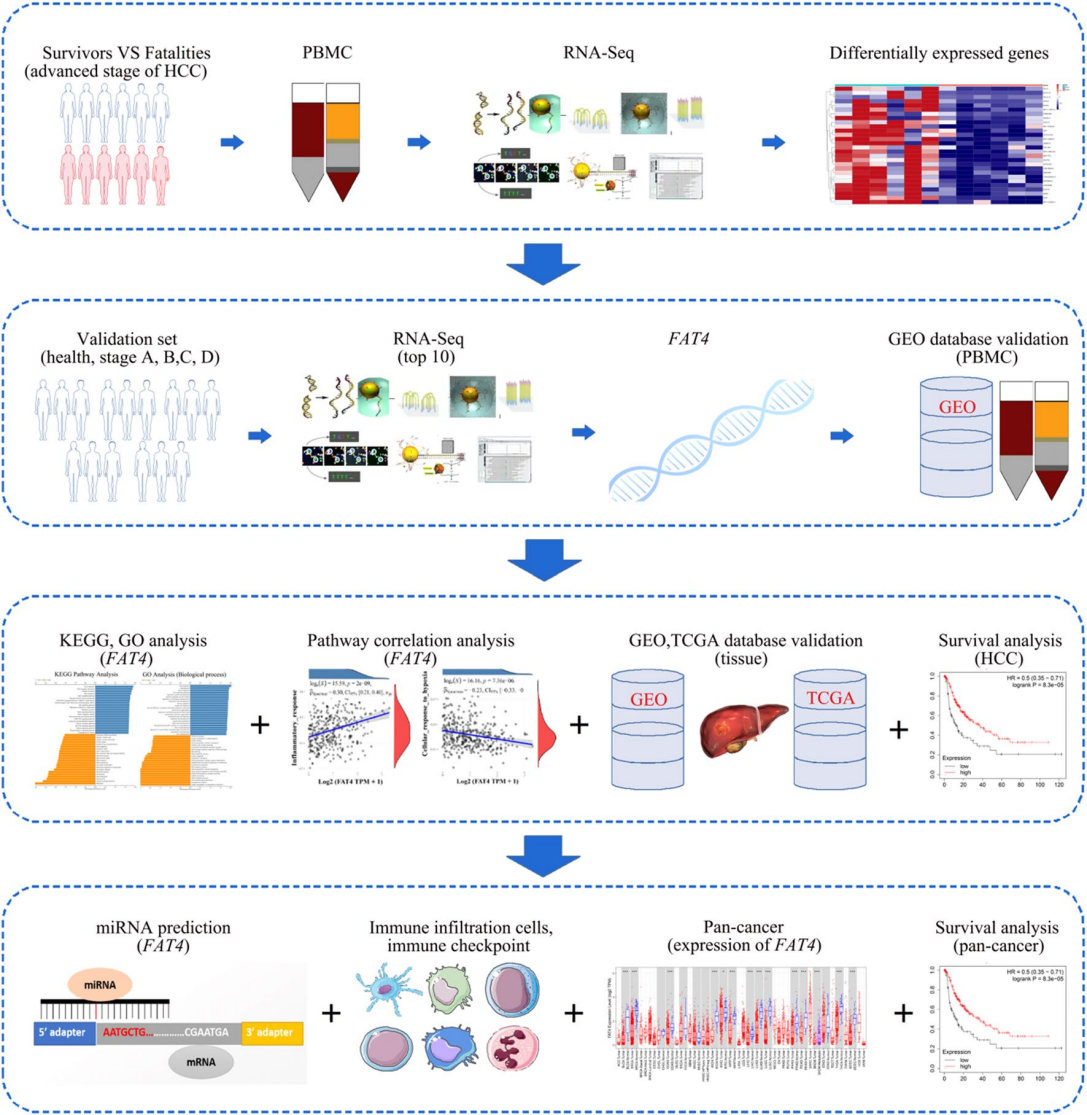
An overview of the experiment. *FAT4*—FAT atypical cadherin 4; GEO—gene expression omnibus; GO—gene ontology; HCC—hepatocellular carcinoma; KEGG—kyoto encyclopedia of genes and genomes; miRNA—microRNA; PBMC—peripheral blood mononuclear cell; RNA-seq—RNA sequencing; TCGA—the cancer genome atlas.

**Figure 2:**
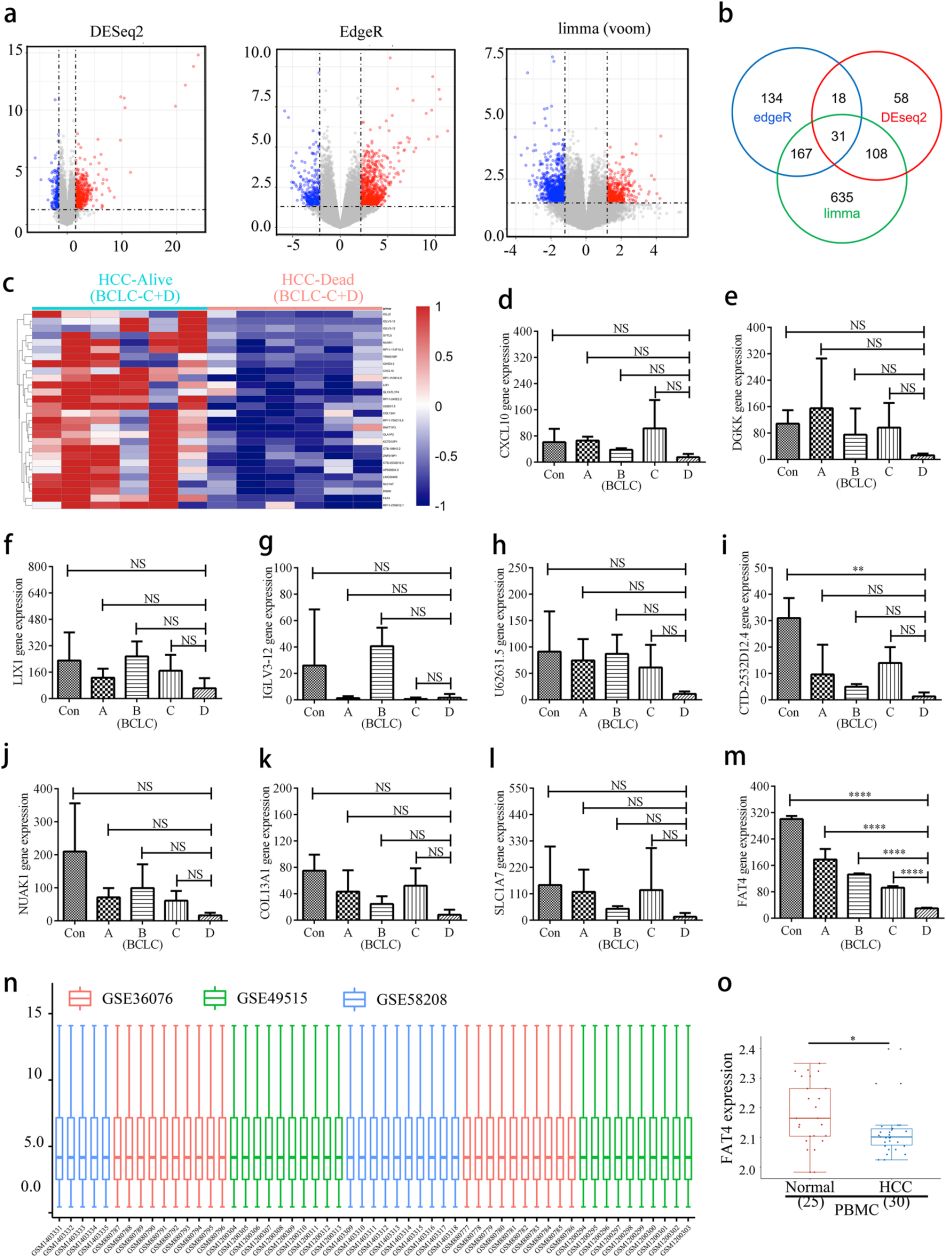
Identification of prognostically relevant genes in PBMCs of patients with HCC. (**a**) Volcano plots showing DEGs between HCC-Alive (*n* = 6) and HCC-Dead (*n* = 6) PBMCs (“DESeq2,” “edgeR,” and “limma” R packages [log fold-change ≥ 2; adjusted *p* value < 0.05]). Red dots represent upregulated genes; blue dots represent downregulated genes. (**b**) Venn diagram comparing DEGs identified using each R package. (**c**) Heatmap showing the relative expression levels of DEGs, in which 31 DEGs were common to all three datasets. (**d**–**m**) RNA-seq validation of selected genes: (**d**) *DGKK*, I *LIX1*, (**f**) *NUAK1*, (**g**) *CXCL10*, (**h**) *FAT4*, (**i**) *U62631.5*, (**j**) *SLC1A7*, (**k**) *COL13A1*, (**l**) *IGLV3-12*, and (**m**) *CTD-2532D12.4*. Statistical analysis was conducted using a one-way analysis of variance. (**n**) Boxplot of GSE36076 (red), GSE49515 (green), and GSE58208 (blue) data normalization. (**o**) *FAT4* expression in PBMCs from patients with HCC and control participants from datasets GSE36776, GSE60502, GSE62232, and GSE36776 from the Gene Expression Omnibus database. BCLC—barcelona clinic liver cancer; Con—control; DEG—differentially expressed gene; *FAT4*—FAT atypical cadherin 4; HCC—hepatocellular carcinoma; NS—not significant; PBMC—peripheral blood mononuclear cell; RNA-Seq—RNA sequencing.

The experimental overview of this study is presented in Fig. 1. A total of 12 samples were analyzed, including six from Barcelona Clinic Liver Cancer (BCLC) stage C death cases, four from BCLC stage C survivors, and two from BCLC stage D survivors. Genes that were not expressed in any of the samples were removed from the PBMCs RNA-Seq data, resulting in the screening of 6004 genes. The PBMC transcriptome confidence intervals of the two patient cohorts were compared using the R packages “DESeq2,” “edgeR,” and “limma” based on screening criteria of *p* < 0.05 and 95% log fold-change cutoff, identifying 499, 894, and 427 differentially expressed genes (DEGs), respectively (Fig. 2a). A Venn diagram showed that 31 DEGs were common to the three datasets (Fig. 2b). DEGs were translated using the GENECODE v22 official annotation file (Fig. 2c). The top ten genes in survivors were *DGKK*, *LIX1*, *NUAK1*, *CXCL10*, *FAT4*, *U62631*.*5*, *SLC1A7*, *COL13A1*, *IGLV3-12*, and *CTD-2532D12.4.*

The RNA-Seq data showed that the mRNA expression of these ten genes was downregulated in the PBMCs of patients with stage D HCC (Fig. 2d,e,f,g,h,i,j,k,l,m). Except for *FAT4*, significant differences were not observed between the expression levels of these genes among all patients^,^ samples. Furthermore, *FAT4* mRNA expression in PBMCs showed correlations with the BCLC stage; that is, patients with more advanced cancer tended to express lower mRNA levels of *FAT4* (Fig. 2m). On analyzing *FAT4* transcript levels, *FAT4* mRNA expression in HCC PBMCs was lower than that in normal PBMCs (Fig. 2n, o).

### Analysis of FAT4 function in HCC

**Figure 3:**
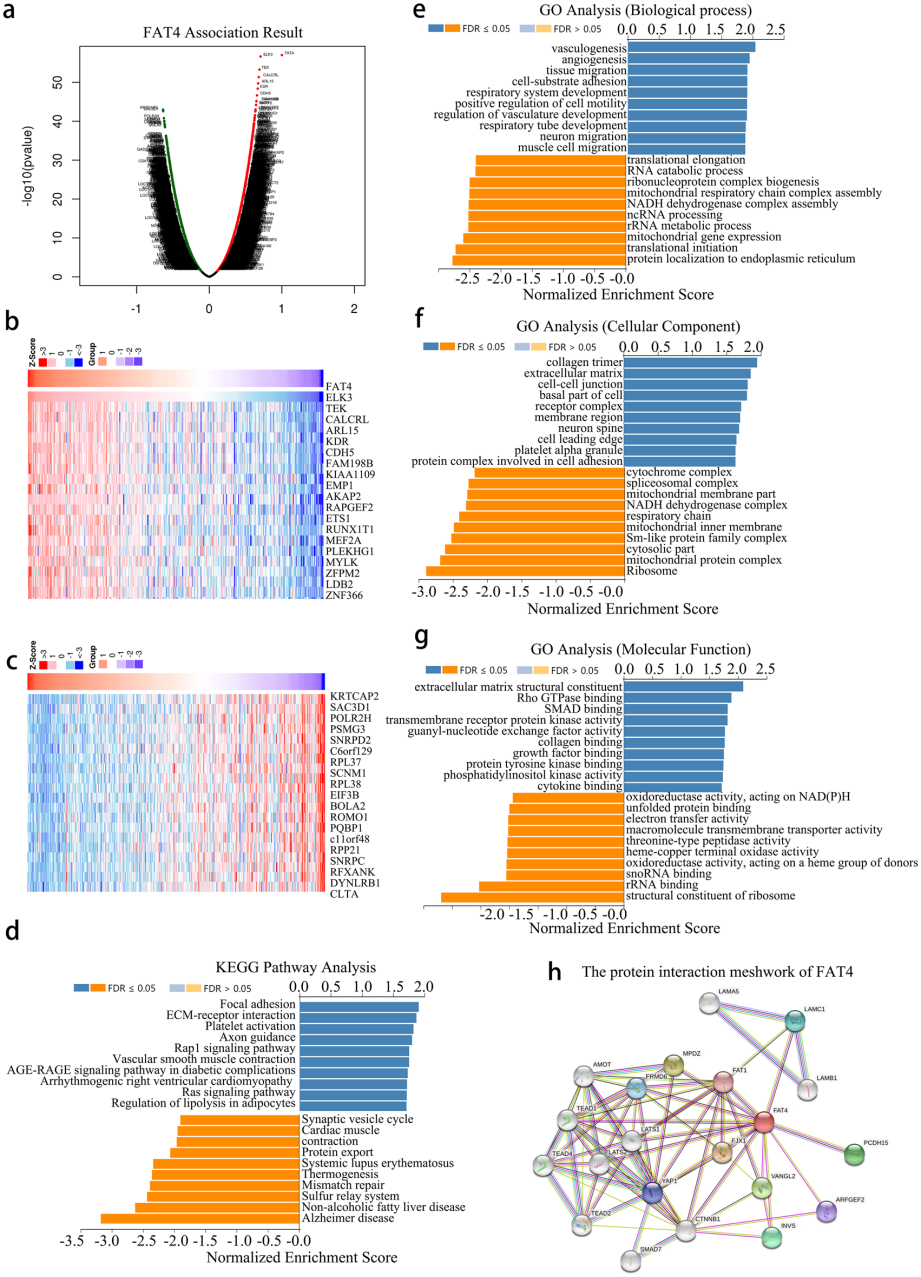
Analysis of *FAT4* function in HCC. (**a**) Volcano plot showing the differential expression of *FAT4*-related genes. (**b**, **c**) Heatmaps showing the top 20 genes positively and negatively correlated with *FAT4*. (**d**) LinkedOmics analysis of *FAT4*-related KEGG pathways in HCC. (**e**–**g**) GO analysis of *FAT4* expression in HCC. (**h**) PPI network of FAT4. *FAT4*—FAT atypical cadherin 4; GO—gene ontology; HCC—hepatocellular carcinoma; KEGG—kyoto encyclopedia of genes and genomes; PPI—protein–protein interaction.

The LinkedOmics database was used to examine *FAT4* expression in The Cancer Genome Atlas (TCGA)-HCC cohort (Fig. 3a). Significant genes that were positively and negatively correlated with *FAT4* are shown in Fig. 3b and c. Kyoto Encyclopedia of Genes and Genomes (KEGG) pathway enrichment analysis showed that *FAT4*-co-expressed genes were primarily involved in focal adhesion, extracellular matrix (ECM)-receptor interactions, platelet activation, axon guidance, the Rap1 signaling pathway, vascular smooth muscle contraction, the AGE-RAGE signaling pathway in diabetes, arrhythmogenic right ventricular cardiomyopathy, the Ras signaling pathway, and lipolysis regulation in adipocytes (Fig. 3d).

With respect to Gene Ontology (GO) analysis, Gene Set Enrichment Analysis (GSEA) indicated that *FAT4*-co-expressed genes were primarily enriched in the biological processes of vasculogenesis, angiogenesis, tissue migration, cell-substrate adhesion, respiratory system development, positive regulation of cell motility, regulation of vasculature development, respiratory tube development, neuron migration, and muscle cell migration (Fig. 3e). Additionally, *FAT4*-co-expressed genes were enriched in various cellular components, namely, collagen trimer, extracellular matrix, cell–cell junction, basal part of the cell, receptor complex, membrane region, neuron spine, cell leading edge, platelet alpha granule, and protein complex involved in cell adhesion (Fig. 3f). Meanwhile, *FAT4*-co-expressed genes were primarily enriched in molecular functions related to extracellular matrix structural constituents, GTPase binding, SMAD binding, transmembrane receptor protein kinase activity, guanyl-nucleotide exchange factor activity, collagen binding, growth factor binding, protein tyrosine kinase binding, phosphatidylinositol kinase activity, and cytokine binding (Fig. 3g). Finally, the STRING database was used to construct a protein–protein interaction (PPI) network of FAT4 (Fig. 3h) and found a possible interaction between FAT4 and YAP1, PDDH15, CTNNB1 through protein interactions.

### Analysis of FAT4-related signaling pathways in HCC

**Figure 4:**
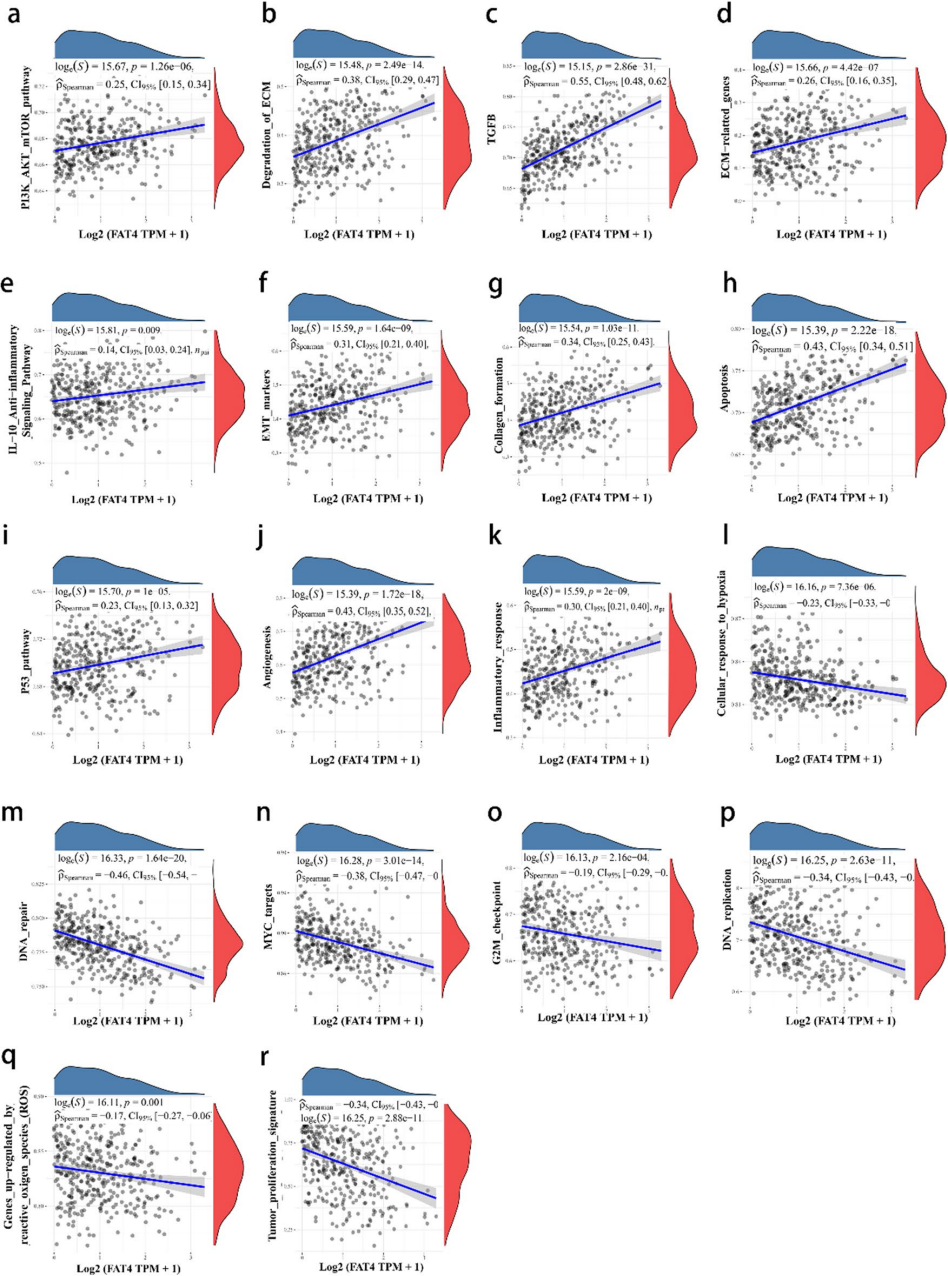
Analysis of *FAT4*-related signaling pathways in HCC. (**a**–**r**) Correlations between *FAT4* expression in HCC and pathway scores analyzed using the R package “GSVA.” The abscissa represents the distribution of the gene expression, and the ordinate represents the distribution of the pathway score. CI—confidence interval; *FAT4*—FAT atypical cadherin 4; HCC—hepatocellular carcinoma.

FAT4 expression was associated with several signaling pathways in HCC, including the PI3K/AKT/mTOR pathway, ECM degradation, transforming growth factor β (TGF-β), ECM-related genes, the IL-10 anti-inflammatory signaling pathway, epithelial–mesenchymal transition (EMT) markers, collagen formation, apoptosis, p53 pathway, angiogenesis, and inflammatory response (Fig. 4a,b,c,d,e,f,g,h,i,j,k). Moreover, *FAT4* expression was negatively correlated with DNA repair, MYC targets, G2M checkpoint, DNA replication, reactive oxygen species-upregulated genes, cellular response to hypoxia, and tumor proliferation signature (Fig. 4l,m,n,o,p,q,r).

### FAT4 expression and prognosis in HCC

**Figure 5:**
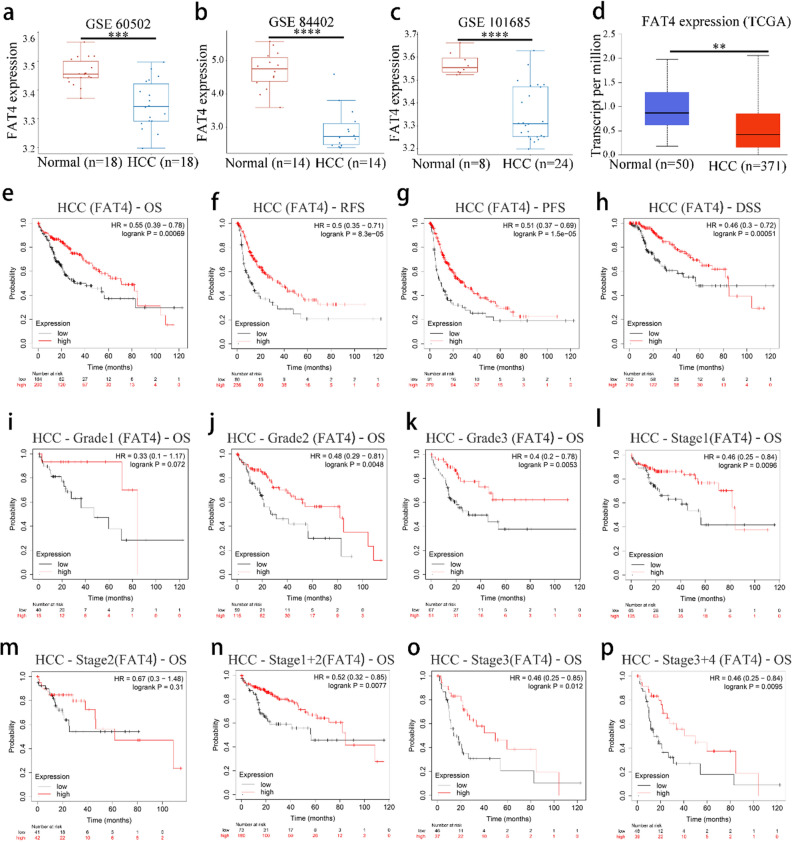
*FAT4* expression and prognosis in HCC. (**a**–**c**) *FAT4* expression in HCC and adjacent non-cancer tissues from datasets (**a**) GSE60502, (**b**) GSE84402, and (**c**) GSE101685 from the Gene Expression Omnibus database. (**d**) *FAT4* expression in HCC and adjacent non-cancer tissues from TCGA database. (**e**–**h**) Kaplan–Meier curves according to *FAT4* expression in HCC: (**e**) OS, (**f**) PFS, (**g**) RFS, and (**h**) DSS. (**i**–**k**) OS curves according to *FAT4* expression, stratified by HCC grade: (**i**) Grade 1, (**j**) Grade 2, and (**k**) Grade 3. (**l**–**p**) OS curves according to *FAT4* expression, stratified by HCC stage: (**l**) Stage 1, (**m**) Stage 2, (**n**) Stage 1 + 2, (**o**) Stage 3, and (**p**) Stage 3 + 4. DSS—disease-specific survival; *FAT4*—FAT atypical cadherin 4; HCC—hepatocellular carcinoma; HR—hazard ratio; OS—overall survival; PFS—progression-free survival; RFS—relapse-free survival; TCGA—the cancer genome atlas. **p < 0.01, ***p < 0.001, ****p < 0.0001.

The Gene Expression Omnibus (GEO) and TCGA analyses showed that *FAT4* mRNA expression was lower in HCC tissues than in normal tissues (Fig. 5a,b,c,d). The associations between *FAT4* expression and various clinicopathological features (stage, race, sex, age, weight, grade, nodal metastasis, *TP53* mutation status, and tumor histology) of HCC were also determined using the UALCAN database. Low *FAT4* expression was found to be significantly associated with stage, race, sex, age, weight, grade, *TP53* mutation status, and tumor histology in patients with HCC (Table S3 in the Supplementary Material). Furthermore, HCC single-cell RNA-Seq data were retrieved from the GEO database, and 11 main clusters were detected (Supplementary Figure 1a). The *FAT4* expression in various clusters is depicted (Supplementary Figure 1b) and was more abundant in endothelial cells than in other groups (Supplemental Figure 1c).

The Kaplan–Meier plotter database was then used to estimate the influence of *FAT4* expression on the prognosis of patients with HCC. The results showed that increased *FAT4* expression was associated with a good prognosis (Fig. 5e,f,g,h). In the subgroup analysis, although the high *FAT4* expression and OS of HCC-Grade 1 patients did not reach statistical significance (Fig. 5i), the high *FAT4* expression in HCC-Grade 2 patients was associated with a longer OS (Fig. 5j). Moreover, HCC-Grade 3 patients with high *FAT4* expression had significantly longer OS (Fig. 5k) and HCC-Stage 1 patients with high *FAT4* expression had longer OS (Fig. 5l). However, in HCC-Stage 2 patients, high *FAT4* expression did lead to longer OS (Fig. 5m). Consistent with the findings observed in HCC-Stage 1 patients, high *FAT4* expression was associated with a good prognosis (longer OS) in patients with HCC-Stage 1 + 2, HCC-Stage 3, and HCC-Stage 3 + 4 (Fig. 5n,o,p). Furthermore, the relationships between distinct clinicopathological features in HCC and *FAT4* expression and prognosis were investigated. High *FAT4* expression was associated with longer PFS in men, women, patients with American Joint Committee on Cancer T1 + T2 tumor categories, patients with vascular invasion, and patients with viral hepatitis (Table S4 in the Supplementary Material). No significant differences in high *FAT4* expression and OS were observed among Caucasian patients, male patients, or patients with vascular invasion (Table S4 in the Supplementary Material).

### Correlation between* FAT4* downregulation and MiR-93-5p upregulation in HCC

To determine the potential regulatory miRNAs of *FAT4*, eight potential miRNA targets were screened using several target gene prediction programs, including miRmap, microT, miRanda, PicTar, and TargetScan (Table S5 in the Supplementary Material). The starBase analysis showed that hsa-miR-17-5p (*r* =  − 0.289, *p* = 1.58e − 08), hsa-miR-20a-5p (*r* =  − 0.248, *p* = 1.37e − 06), hsa-miR-93-5p (*r* =  − 0.271, *p* = 1.24e − 07), and hsa-miR-193a-3p (*r* =  − 0.182, *p* = 4.35e − 04) were inversely correlated with *FAT4* expression (Figure Supplemental Figure 2a). The expression levels of the above miRNAs were evaluated in normal and HCC samples using TCGA (Supplemental Figure 2b,c,d,e,f,g,h,i). Collectively, the expression levels of hsa-miR-17-5p, hsa-miR-20a-5p, and hsa-miR-93-5p were significantly higher in HCC tissue than in normal control tissue and negatively correlated with *FAT4* expression, which supported our hypothesis.

Next, the prognostic value of hsa-miR-17-5p, hsa-miR-20a-5p, and hsa-miR-93-5p in HCC were analyzed using Kaplan–Meier curves. The results showed that patients with HCC with low hsa-mir-204-5p levels had better OS than those with high levels (Supplemental Figure 2j,k,l). The binding site between hsa-miR-93-5p and FAT4 in the 3'-untranslated region is shown in Supplemental Figure 2m. These findings suggest that hsa-miR-93-5p may function as a *FAT4*-regulated miRNA in HCC.

**Figure 6:**
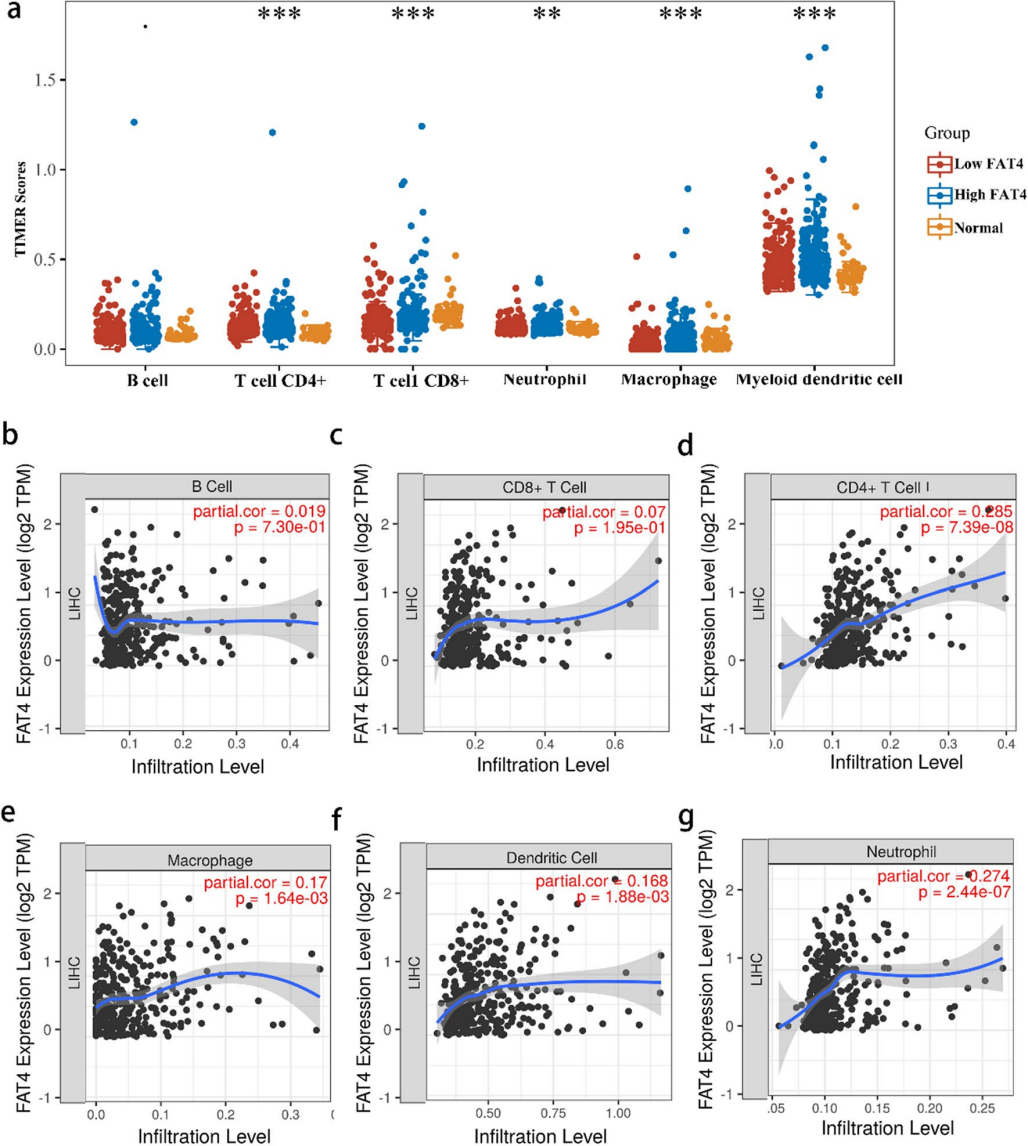
Association between *FAT4* expression and infiltrating immune cells in HCC. (**a**) TIMER scores of various immune cells in HCC tissues with high or low *FAT4* expression and normal controls. (**b**–**g**) Correlation of *FAT4* expression with the infiltration level of (**b**) B cells, (**c**) CD8 + T cells, (**d**) CD4 + T cells, (**e**) macrophages, (**f**) dendritic cells, and (**g**) neutrophils in HCC. *FAT4*—FAT atypical cadherin 4; HCC—hepatocellular carcinoma; LIHC—liver hepatocellular carcinoma; TIMER—tumor immune estimation resource.

### Association between *FAT4* expression and immune infiltrating cells in HCC

The distribution of *FAT4* expression scores in HCC was analyzed using “immunedeconv.” The results showed that the Tumor Immune Estimation Resource (TIMER) scores of CD4 + /CD8 + T cells, neutrophils, macrophages, and dendritic cells were significantly increased in HCC tissues with high *FAT4* expression compared to that in HCC tissues with low *FAT4* expression (Fig. 6a).

Additionally, the association between *FAT4* expression in HCC and immune infiltration was explored using the TIMER database (Fig. 6b,c,d,e,f,g). Significant positive correlations were detected between the expression of *FAT4* and the infiltration levels of CD4 + T cells (*r* = 0.285,* p* = 7.39e − 08), macrophages (*r* = 0.17, *p* = 1.64e − 03), neutrophils (*r* = 0.274, *p* = 2.44e − 07), and dendritic cells (*r* = 0.168, *p* = 1.88e − 03).

Although *FAT4* was found associated with prognosis and several types of infiltrating immune cells in HCC, the association between *FAT4* and immune markers remains unknown. Hence, the associations between the expression of *FAT4* and markers of immune cell subtypes in HCC were analyzed using the TIMER and Gene Expression Profiling Interactive Analysis 2 (GEPIA2) databases (Table S6 in the Supplementary Material). *FAT4* expression was associated with markers of T-regulatory cells, monocytes, M1/M2 macrophages, T helper 1 cells, and mast cells in HCC. Furthermore, *FAT4* expression was associated with markers of tumor-associated macrophages, such as CCL2, CD68, and IL-10. These findings confirm the relationship between *FAT4* and tumor-associated macrophage infiltration. Additionally, relationships were observed between *FAT4* expression and CD4 + T cells (CD86) and dendritic cells (BDCA1 [CD1C], BDCA4 [NRP1], and CD11c [ITGAX]) (Table S6 in the Supplementary Material).

### Relationship between immune checkpoints and *FAT4* expression in HCC

**Figure 7:**
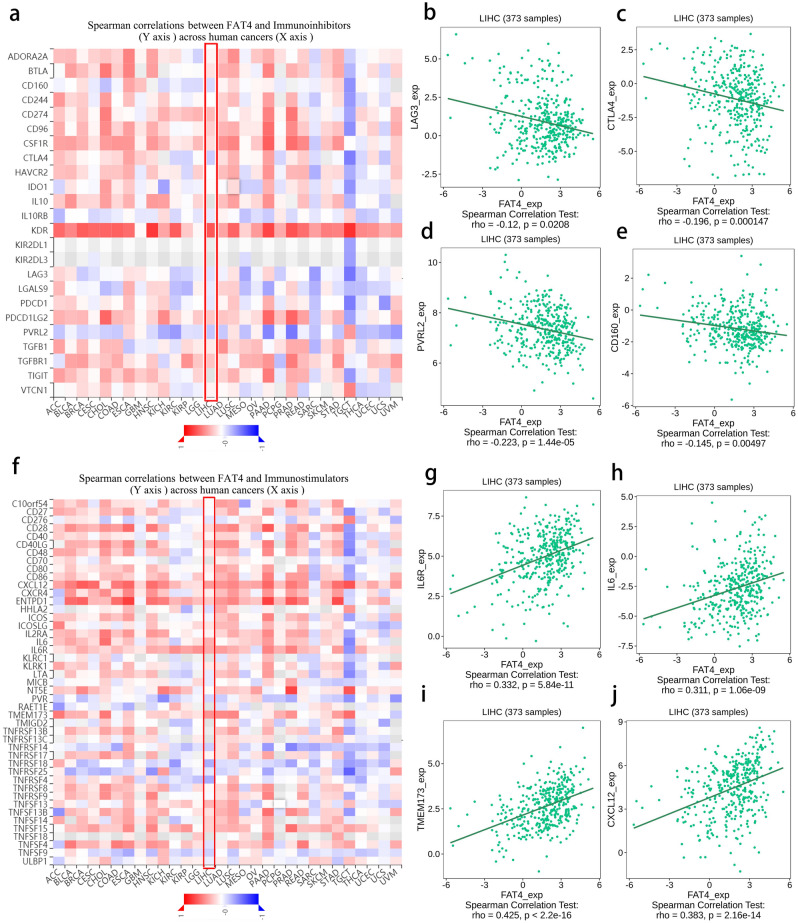
Relationship between *FAT4* expression and immune checkpoints in HCC. (**a**) Heatmap showing the correlation between *FAT4* expression and immunoinhibitor expression in human cancers. *FAT4* expression was negatively correlated with (**b**) *LAG3*, (**c**) *CTLA4*, (**d**) *PVRL2*, and (**e**) *CD160* expression in HCC. (**f**) Heatmap showing the correlation between *FAT4* expression and immunostimulator expression in human cancers. *FAT4* expression was positively correlated with (**g**) *IL6R*, (**h**) *IL6*, (**i**) *TMEM173*, and (**j**) *CXCL12* expression in HCC. *FAT4*—FAT atypical cadherin 4; HCC—hepatocellular carcinoma; LIHC—liver hepatocellular carcinoma.

*FAT4* expression was significantly and negatively correlated with several immunoinhibitors in HCC, including *LAG3*, *CTLA4*, *CD160*, and *PVRL2* (*p* < 0.05; Fig. 7a,b,c,d,e). A relationship was also observed between the expression of *FAT4* and that of several immunostimulators. For example, *FAT4* expression was positively correlated with the expression of *IL6R*, *IL6*, *TMEM173*, and *CXCL12* (*p* < 0.05; Fig. 7f,g,h,I,j). These findings indicate that *FAT4* expression is significantly correlated with immune infiltration in HCC.

### *FAT4* expression and prognostic role in pan-cancer samples

**Figure 8:**
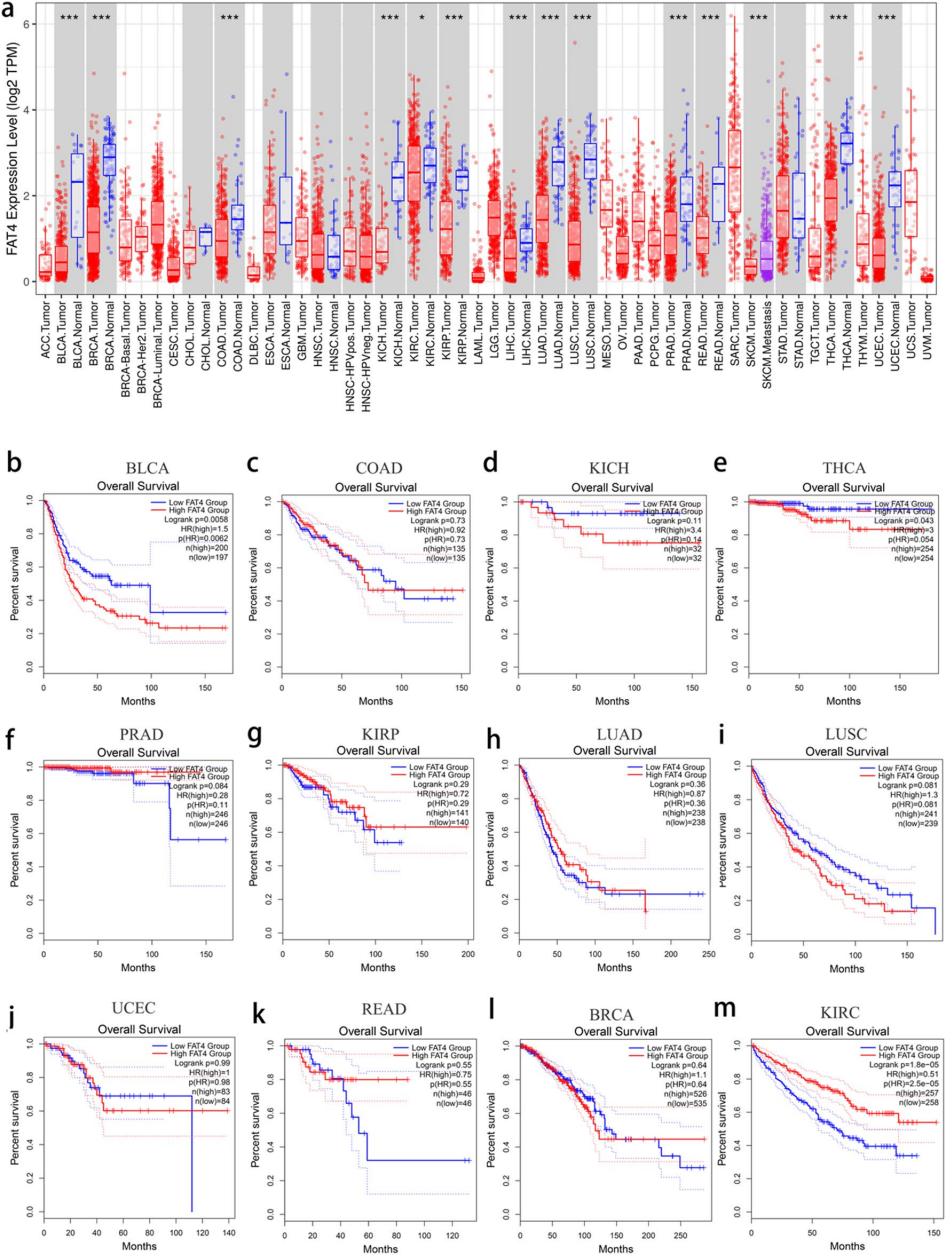
*FAT4* expression and prognostic role in pan-cancer samples. (**a**) *FAT4* expression examined in different cancer types using the TIMER database. Prognosis of *FAT4* in (**b**) BLCA, (**c**) COAD, (**d**) KICH, (**e**) THCA, (**f**) PRAD, (**g**) KIRP, (**h**) LUAD, (**i**) LUSC, (**j**) UCEC, (**k**) READ, (**l**) BRCA, and (**m**) KIRC examined using the GEPIA2 database. BLCA—bladder urothelial carcinoma; BRCA—breast invasive carcinoma; COAD—colon adenocarcinoma; *FAT4*—FAT atypical cadherin 4; GEPIA2—gene expression profiling interactive analysis 2; HCC—hepatocellular carcinoma; HR—hazard ratio; KICH—kidney chromophobe; KIRC—kidney renal clear cell carcinoma; KIRP—kidney renal papillary carcinoma; LIHC—liver hepatocellular carcinoma; LUAD—lung adenocarcinoma; LUSC—lung squamous cell carcinoma; PRAD—prostate adenocarcinoma; READ—rectal adenocarcinoma; THCA—thyroid carcinoma; TIMER—tumor immune estimation resource; UCEC—uterine corpus endometrial carcinoma.

*FAT4* expression was analyzed in various cancers using the TIMER database. The results showed that *FAT4* was significantly upregulated in 13 cancer types compared with that in the normal samples (Fig. 8a). Survival analysis of *FAT4* in these tumors was subsequently performed (Fig. 8b,c,d,e,f,g,h,i,j,k,l,m). Patients with bladder urothelial carcinoma and thyroid carcinoma with upregulated *FAT4* had a good prognosis (Fig. 8a,e), whereas patients with kidney renal clear cell carcinoma with downregulated *FAT4* had a poor prognosis (Fig. 8m).

## Discussion

Serum and plasma levels of specific oncogenes in patients with various cancer types are commonly elevated; thus, the circulating levels of these oncogenes have been used for cancer classification and prognosis^15–17^. However, genes expressed in serum are derived from various tissues, and their levels vary considerably. In contrast, genes expressed in PBMCs are derived from a single source and consist of circulating immune cells, such as monocytes, T lymphocytes, B lymphocytes, and natural killer cells^9^. PBMC gene expression has recently been shown to have important clinical value in diagnosing and predicting the prognosis of myriad malignant tumor types^18–20^. Therefore, finding new non-invasive biomarkers has become important for diagnostic and prognostic evaluations of HCC. Therefore, PBMCs may also represent a “surrogate” for evaluating the prognosis of patients with HCC. Thus, it overrides the disadvantages of liver biopsy, which is currently the most reliable method available for diagnosing HCC, in terms of invasiveness with potential complications and limited clinical applications^21^.

In this study, RNA-Seq was used to comprehensively analyze the mRNA expression of PBMCs in patients with advanced HCC who had either survived or died. RNA-Seq analysis showed that the expression levels of *DGKK*, *LIX1*, *NUAK1*, *CXCL10*, and *FAT4* in the survival group were significantly higher than those in the death group. We further verified the expression of the top ten genes in the PBMCs of the HCC survival group and found that the expression levels of *FAT4* mRNA in PBMCs were closely associated with HCC stage. Hence, *FAT4* mRNA levels in PBMCs may represent a potential biomarker and therapeutic target of HCC.

*FAT4* was first identified as a tumor suppressor in a mouse mammary epithelial cell line^22^. Accumulating evidence suggests that *FAT4* downregulation is involved in the pathogenesis of multiple malignancies, is inversely correlated with tumor grade, and exerts tumor-suppressor effects^23–25^. Consistent with previous findings, we found that it was decreased in several tumors, suggesting that *FAT4* may exhibit tumor suppressor effects in various tumors. Furthermore, patients with kidney renal clear cell carcinoma and high *FAT4* expression exhibited a good prognosis. On investigating the function of *FAT4* in HCC, we found that FAT4 was primarily involved in biological processes related to cell motility and that *FAT4* expression was significantly positively correlated with the PI3K/AKT/mTOR pathway, ECM degradation, transforming growth factor β, ECM-related genes, and IL-10 anti-inflammatory signaling pathway in HCC. Thus, FAT4 may have a tumor suppressor role in HCC by inhibiting tumor cell invasion and metastasis. Indeed, FAT4 has been shown to have an important role in inhibiting EMT and human gastric cancer cell proliferation^26^. Meanwhile, in colorectal cancer, FAT4 has been found to regulate PI3K activity in the PI3K/AKT/mTOR signaling pathway, inhibit tumor growth, and contribute to preventing EMT^25^. Based on these findings, we speculated that *FAT4* may be abnormally expressed in HCC tissues. Aligning with this speculation, we found that *FAT4* expression in HCC tissues was significantly lower than that in normal liver tissues. Moreover, the OS of patients with HCC and high *FAT4* expression was prolonged, further suggesting that *FAT4* has tumor suppressor effects in HCC and may serve as a novel prognostic biomarker.

Increasing evidence suggests that the tumor microenvironment and immune infiltration greatly impact the development and progression of tumors^27,28^. Immunotherapies that enhance the ability of the immune system to target and eliminate tumor cells have been shown to be effective in some patients with advanced HCC^7^. However, the levels of immune cell infiltration and immune checkpoint expression vary among tumor types, resulting in different clinical responses in patients^28^. Therefore, additional HCC immunotherapy targets and precise prognostic markers of immune infiltration are urgently required. Based on our findings, we further evaluated the correlation between *FAT4* and immune cell infiltration in HCC. *FAT4* expression was significantly and positively correlated with the infiltration of neutrophils, macrophages, dendritic cells, and CD4 + T cells. At the forefront of host defenses, macrophages are significant contributors to tumor immunity^29^. Additionally, CD4 + T helper cells are major players in humoral immunity, pathogen protection, and autoimmunity induction^30^. Dendritic cells can induce specific or non-specific immune responses and elicit tumor-killing and antiviral effects in patients^31^. Neutrophils can also infiltrate tumors, while differences in the tumor microenvironment may serve as the driving, or reaction, force of tumor progression^32^. Collectively, these results suggest that *FAT4* may exert tumor suppressor effects by promoting the infiltration of tumor-associated immune cells.

Additionally, immune checkpoints significantly impact the tumor immune response^33^. Immune checkpoints include both stimulatory and inhibitory checkpoints. Unlike the stimulatory checkpoints, which promote the body’s immune response, inhibitory checkpoints are protective sites that prevent the body from exerting immune responses and can reduce autoimmune responses; therefore, they are often used by tumor cells to evade the immune system^34,35^. We found that *FAT4* expression was positively correlated with the expression of most immune-activating factors and negatively correlated with the expression of certain immunosuppressive factors, indicating that *FAT4* contributes to the recruitment and regulation of immune cell infiltration in HCC.

Considering that miRNAs contribute to gene expression regulation^36^, we sought to identify upstream regulatory miRNAs of *FAT4.* After a comprehensive analysis, miRNA-93-5p was selected as a potential miRNA that binds with *FAT4*. A previous study showed that miR-93-5p enhances the proliferation, migration, and invasion of gastric cancer cells by activating the Hippo pathway, making it a potential diagnostic and therapeutic target for gastric cancer^37^. Moreover, in pancreatic cancer, miR-93 diminishes *PTEN* expression by inhibiting PI3K/Akt and promoting cancer cell migration, invasion, and proliferation^38^. These results suggest that miRNA-93-5p may act as an upstream regulatory miRNA of *FAT4* in HCC. Moreover, miR-93-5p has been found to inactivate infiltrating CD8 + T cells and induce immune evasion by targeting multiple regulators and modulating the bivalence of H3K4me3/H3K27me3^39^. In addition, myeloid-derived suppressor cells exosomes of miR-93-5p inhibited the differentiation of Th1 and Th17 cells in vitro, which had immunosuppressive effects^40^. These findings further confirm that *FAT4* may play a tumor-suppressive role by promoting the infiltration of tumor-associated immune cells. In addition, we found a possible interaction between *FAT4* and YAP1 through protein interactions. Previous animal experiment with a BC mouse model established using 4T1 cells has indicated that inhibiting YAP suppressed tumor weight and volume and inhibited inflammatory infiltration. Inhibition of YAP decreased the M2/M1 macrophage ratio and Treg cell ratio and increased the CD8 + and CD4 + T cell ratio^41^. These results suggest that *FAT4* may exert anticancer effects by inhibiting YAP and thus promoting immune infiltration. In addition, we found that the expression of *FAT4* was associated with the TGF-β signaling pathway in HCC. TGF-β stimulation of tumor cells was shown to increase SHP1 phosphatase activity in an AKT-Smad3-dependent manner, decrease IFNγ-mediated tyrosine phosphorylation of JAK1/2 and STAT1, and inhibit STAT1-dependent immune evasion-associated molecule expression, such as PD-L1, IDO1, herpesvirus entry medium (HVEM), and galectin-9 (Gal-9). In a mouse model of lung cancer, dual blockade of TGF-β and PD-L1 was associated with stronger anti-tumor activity and prolonged survival compared to anti-PD-L1 therapy alone^42^. These findings suggest that *FAT4* may promote T lymphocyte infiltration by affecting the TGF-β signaling pathway, thereby enhancing anti-tumor immunity.

The present study has certain limitations. First, the role of *FAT4* has been validated in a small subset of patients. Although *FAT4* mRNA and protein expression levels were further validated using data from multiple public databases, rigorous validations on a larger scale are needed. In addition, in vivo and in vitro experiments are required to validate the specific function of hsa-miR-93-5P/*FAT4* in HCC. Finally, the molecular mechanisms associated with *FAT4* and the ability of *FAT4* to regulate tumor-infiltrating cells and affect the prognosis of patients with HCC must be further explored. Therefore, future experiments should investigate the specific mechanisms by which *FAT4* regulates immune cell infiltration to identify novel targets for tumor immunotherapy.

## Conclusions

To the best of our knowledge, this is the first study to report that *FAT4* expression is downregulated in the PBMCs of patients with HCC, associated with prognosis and significantly correlated with diverse clinicopathological characteristics. Additionally, *FAT4* expression was associated with the infiltration of anti-tumor-related immune cells and the expression of immune activators. Moreover, we identified the hsa-miR-93-5p/*FAT4* axis as the upstream regulatory mechanism of *FAT4* in HCC. These findings provide an important understanding of the dysregulation of *FAT4* in HCC and suggest that *FAT4* may have important functions in the tumor immune microenvironment.

## Materials and methods

### Ethical statement

All procedures involving human participants were performed in accordance with the ethical standards of the institutional and/or national research committee and with the 1964 Declaration of Helsinki and its later amendments or comparable ethical standards. The study design was approved by the Ethics Committee of Shenzhen Hospital of Traditional Chinese Medicine (approval number 2018/58). Written informed consent was obtained from each subject.

### Patient samples

HCC was staged according to the BCLC staging system as follows: stage 0 (very early), stage A (early), stage B (intermediate), stage C (advanced), and stage D (terminal). Different treatments are indicated for each stage^21^. Between January 2018 and December 2019, 12 patients with BCLC stage C or D HCC were recruited for this study (Table S1 in the Supplementary Material for detailed demographic data). Eight milliliters of peripheral blood was collected from each patient utilizing EDTA anticoagulant vacuum angiography. Subsequently, PBMCs were extracted from whole blood via density gradient centrifugation. RNA was extracted using TRIzol reagent (Invitrogen, Carlsbad, CA, USA) according to the manufacturer’s instructions. RNA quality was assessed by measuring the absorbance at 260 nm (A260) and 280 nm (A280) using a NanoDrop ND-1000 (Thermo Fisher Scientific, Waltham, MA). Additionally, RNA integrity was determined based on the RNA integrity number (RIN) using Agilent 2100 RIN Beta Version Software (Agilent Technologies, Santa Clara, CA, USA). Patients were followed up regularly. Between January 2020 and January 2021, 15 patients were recruited into the validation cohort: healthy (*n* = 3), stage A (*n* = 3), stage B (*n* = 3), stage C (*n* = 3), and stage D (*n* = 3) (Table S2 in the Supplementary Material for detailed demographic data). Blood sampling, RNA extraction, and RNA-sequencing were performed using the same method as that applied for the HCC cohort.

### RNA-seq library

RNA samples were prepared using a total of 5 g RNA per sample as input material. Ribosomal RNA was removed using the Epicentre Ribozero rRNA Removal Kit (Epicentre, New York, NY, USA), and rRNA-free residue was cleaned using ethanol precipitation. Linear RNA was then digested with 3 U of RNase R (Epicentre, USA) per g of RNA. Sequencing libraries were generated using a NEBNext Ultra Directional RNA Library Prep Kit for Illumina (NEB, USA) according to the manufacturer's instructions. Fragmentation was performed in NEBNext First Strand Synthesis Reaction Buffer utilizing divalent cations at elevated temperatures. Random hexamer primers and M-MuLV Reverse Transcriptase (RNaseH-) were used to create first-strand cDNA. Subsequently, DNA polymerase I and RNase H were used to generate second-strand cDNA synthesis. dNTPs with dTTP were substituted by dUTP in the reaction buffer. Exonuclease/polymerase activities were used to convert the remaining overhangs into blunt ends. To prepare for hybridization, NEBNext adaptors with hairpin loop structures were ligated after adenylation of the 3’ ends of DNA fragments. The library fragments were purified using AMPure XP technology (Beckman Coulter, Beverly, USA) to select cDNA fragments of primarily 250–300 bp in length. Prior to PCR, 3 L of USER Enzyme (NEB, USA) was applied with size-selected, adaptor-ligated cDNA for 15 min at 37 °C, followed by 5 min at 95 °C. PCR was then carried out using Phusion High-Fidelity DNA Polymerase, universal PCR primers, and indexed primers. Finally, the products were purified (AMPure XP system) and library quality was evaluated using an Agilent Bioanalyzer 2100 system. Paired-end sequencing (2 × 150 bp) was performed using an Illumina HiSeq X TEN Reagent Kit (NEB, USA).

### Identification of DEGs

Raw RNA-Seq data were filtered using SOAPnuke (version 1.0.1). High-quality clean reads were aligned with the human transcriptome and HG19 genome using TopHat2 (version 2.0.7). Genes that were not present in any of the samples were eliminated. The R packages “DESeq2,” “edgeR,” and “limma” were used to screen DEGs. A Venn diagram was constructed to compare DEGs in each group using the R package “Venny2.1.”

### Gene expression analysis

*FAT4* expression data and clinical information from datasets GSE60502, GSE84402, GSE101685, GSE36076, GSE49515, and GSE58208 were downloaded from the Gene Expression Omnibus (GEO) database and analyzed for *FAT4* expression. Raw data were downloaded as MINiML files. Box plots were drawn using “boxplot,” and principal component analysis (PCA) plots were drawn using “ggord.” The UALCAN database (http://ualcan.path.uab.edu/)^43^ was used to investigate *FAT4* expression in HCC and to elucidate the relationship between *FAT4* expression and various clinicopathological features of HCC (stage, race, sex, age, weight, grade, nodal metastasis, *TP53* [tumor protein p53] mutation status, and tumor histology). Additionally, the TIMER database (https://cistrome.shinyapps.io/timer/)^44^ was used to analyze the mRNA levels of *FAT4* in several cancers.

### Single-cell expression pattern and subcellular localization of FAT4 in HCC

Data were obtained from the GSE112271 dataset (https://www.ncbi.nlm.nih.gov/geo/) (Supplementary Material)^45^ and analyzed as described above^46^. Briefly, the “Seurat” package was used to generate objects and filter out poor quality cells, and a standard data preprocessing procedure was applied. The percentages of gene counts, cell counts, and mitochondrial content were calculated, and genes were filtered based on the following criteria: genes detected in fewer than 3 cells and cells detected with fewer than 200 genes. To normalize each cell, we scaled the UMI counts using a scale factor of 10,000. After performing a logarithmic transformation of the data, corrected normalized data metrics were applied to the standard analysis using the ScaleData function in Seurat (v3.0.2). The first 2000 variable genes were extracted for PCA. We performed cell clustering using the FindClusters function (resolution = 0.5) implemented in the Seurat R package to explore the correlation between *FAT4* and different cell populations in HCC.

### Survival analysis

*FAT4* expression levels affecting the clinical outcomes of patients with HCC were estimated using Kaplan–Meier survival curves (http://kmplot.com/analysis/)^47^. The GEPIA2 database (http://gepia.cancer-pku.cn/index.html)^48^ was used to assess the effect of *FAT4*-related genes (mRNA, miRNA, and protein) on survival and the potential prognostic value of *FAT4* in 12 cancer types.

### GSEA and PPI network construction

The LinkedOmics database (http://linkedomics.org/login.php)^49^ was utilized to identify co-expressed genes of *FAT4* in HCC. Additionally, GSEA was used to perform a KEGG enrichment analysis of *FAT4* signaling pathways and GO analysis of *FAT4* in HCC. A PPI network of *FAT4* was constructed using the STRING database (https://string-db.org/). The detailed analysis method was previously described^50,51^.

### Analysis of FAT4 signaling pathways in HCC

RNA expression (level 3) profiles and corresponding clinical information on HCC were downloaded from TCGA (https://portal.gdc.com). Data were analyzed using the R package “GSVA.” The selected parameter was “ssgsea.” Correlations between *FAT4* and pathway scores were analyzed using Spearman’s correlation.

### Prediction of upstream MiRNAs regulating *FAT4*

starBase (v2.0) was used to predict miRNAs that could potentially bind *FAT4*^52^. Upstream miRNAs of *FAT4* were predicted using miRmap, microT, miRanda, PicTar, and TargetScan. starBase was also used to perform expression correlation analysis between the predicted miRNAs and *FAT4* in HCC.

### Immune infiltration analysis

Software packages “ggplot2” and “pheatmap” for R (version 4.0.3) (R Foundation for Statistical Computing) were used to assess the immune score of various immune cells in HCC tissues with high or low *FAT4* expression as well as in normal tissues. TIMER was used to examine the relationship between somatic copy number alterations in *FAT4* and the infiltration levels of B cells, CD4 + /CD8 + T cells, neutrophils, macrophages, and dendritic cells. The correlation modules in GEPIA2 and TIMER were used to estimate the Spearman’s correlation coefficient between *FAT4* expression and markers of tumor-infiltrating immune cells in HCC^44,48^. Additionally, the “immunostimulator” and “immunoinhibitor” modules in the Tumor and Immune System Interaction Database (TISIDB) (http://cis.hku.hk/TISIDB)^53^ were used to evaluate the relationship between *FAT4* expression and immune checkpoints.

### Statistical analysis

All statistical analyses were conducted using R (version 4.0.1) and SPSS (version 22.0). PCA and Pearson’s correlation analysis were performed to evaluate sample similarity. Spearman’s correlation analysis was used to analyze correlations. Statistical differences were examined using a *t* test or one-way analysis of variance. All statistical tests were two-sided. Statistical significance was set at *p* < 0.05.

### Supplementary Information

Supplementary Information.

## Data Availability

The datasets generated and/or evaluated during the current study are available at NCBI project PRJNA909469 (https://www.ncbi.nlm.nih.gov/bioproject/PRJNA909469).
