# Supplementary material for: FAT4 expression in peripheral blood mononuclear cells is associated with prognosis and immune cell infiltration in hepatocellular carcinoma
Source: Sci Rep. 2023 Sep 21;13:15735. doi: 10.1038/s41598-023-42560-w (PMC10514079; doi:10.1038/s41598-023-42560-w)
Supplement: Supplementary file 1 — Supplementary Information. [file 41598_2023_42560_MOESM1_ESM.docx]

# *FAT4* Expression in Peripheral Blood Mononuclear Cells Is Associated with Prognosis and Immune Cell Infiltration in Hepatocellular Carcinoma

Jing Li,^1,2,3^ Minling Lv,^1,2^ Qi [Huang](mailto:janethhq@163.com;),^1,2^ Rui Hu,^1,2,3^ Xin Zhong,^1,2^ Xinfeng Sun,^1,2^ Wenxing Feng,^1,2^ Zhiyi Han,^1,2^ MengQing Ma,^1,2^ Wei Zhang,^1,2^ and Xiaozhou Zhou^1,2,*^

| **Characteristic** | **live** | **Dead** |
| --- | --- | --- |
| Number | 6 | 6 |
| Gender |  |  |
| male | 2 | 1 |
| female | 4 | 5 |
| Age (mean ± SD) | 55.17±4.4 | 54.33±4.849 |
| BCLC |  |  |
| C | 4 | 5 |
| D | 2 | 1 |
| child_pugh_classification_grade |  |  |
| A | 3 | 2 |
| B | 1 | 1 |
| C | 2 | 3 |
| HBV |  |  |
| Yes | 6 | 6 |
| No | 0 | 0 |
| Metastasis |  |  |
| Yes | 4 | 5 |
| No | 2 | 1 |
| Cirrhosis |  |  |
| Yes | 4 | 5 |
| No | 2 | 1 |

**Supplementary Table 1. Demographics**

**Supplementary Table 2. Demographics of the validation cohort**

| Characteristic | BCLC (stage 0) | BCLC (stage A) | BCLC  (stage B) | BCLC  (stage C) | BCLC  (stage D) |
| --- | --- | --- | --- | --- | --- |
| Number | 3 | 3 | 3 | 3 | 3 |
| Gender |  |  |  |  |  |
| male | 3 | 3 | 1 |  | 3 |
| female | 0 | 0 | 2 |  | 0 |
| Age (mean ± SD) | 30±12 | 58±13 | 63±11 | 43±4 | 44±8 |
| HBV |  |  |  |  |  |
| Yes | 0 | 3 | 2 |  | 3 |
| child_pugh_classification_grade |  |  |  |  |  |
| A | 0 | 3 | 3 | 3 | 1 |
| B | 0 | 0 | 0 | 0 | 1 |
| C | 0 | 0 | 0 | 0 | 1 |
| No | 3 | 0 | 1 | 0 | 0 |
| Metastasis |  |  |  |  |  |
| Yes | 0 | 1 | 0 | 0 | 0 |
| No | 3 | 2 | 3 | 3 | 3 |
| Cirrhosis |  |  |  |  |  |
| Yes | 0 | 2 | 3 | 0 | 1 |
| No | 3 | 1 | 0 | 3 | 2 |

**Supplementary Table 3. FAT4 expression in LIHC of different Sample types in UALCAN**

| TCGA samples | Transcript per million | | | Comparison | P |
| --- | --- | --- | --- | --- | --- |
| **Sample types** | low | median | high |  |  |
| **Stages** |  |  |  |  |  |
| Stagel  (n=168) | 0 | 0.459 | 1.966 | Normal-vs-Stage1 | **4.54E-03** |
| Stage2  (n=84) | 0.004 | 0.247 | 1.489 | Normal-vs-Stage2 | **5.99E-04** |
| Stage3  (n=82) | 0 | 0.343 | 1.838 | Normal-vs-Stage3 | **1.91E-02** |
| Stage4  (n=6) | 0.094 | 0.251 | 0.96 | Normal-vs-Stage4 | **1.37E-02** |
| **Race** |  |  |  |  |  |
| Caucasian  (n=177) | 0 | 0.59 | 2.372 | Normal-vs-Caucasian | **7.18E-02** |
| African-american  (n=17) | 0.051 | 0.399 | 1.855 | Normal-vs-AfricanAmerican | **2.37E-02** |
| Asian  (n=157) | 0 | 0.294 | 1.611 | Normal-vs-Asian | **3.52E-05** |
| **Gender** |  |  |  |  |  |
| Male  (n=245) | 0 | 0.393 | 1.966 | Normal-vs-Male | **2.38E-04** |
| Female  (n=117) | 0.01 | 0.472 | 2.545 | Normal-vs-Female | **5.30E-02** |
| **Age** |  |  |  |  |  |
| 21-40 Yrs  (n=27) | 0.01 | 0.507 | 0.965 | Normal-vs-Age(21-40Yrs) | 1.75E-01 |
| 41-60 Yrs  (n=140) | 0 | 0.382 | 0.819 | Normal-vs-Age(41-60Yrs) | **1.66E-03** |
| 61-80 Yrs  (n=181) | 0 | 0.412 | 0.882 | Normal-vs-Age(61-80Yrs) | **1.24E-03** |
| 81-100 Yrs  (n=10) | 0.07 | 0.744 | 1.449 | Normal-vs-Age(81-100Yrs) | 9.05E-01 |
| **Weight** |  |  |  |  |  |
| Normal weight  (n=154) | 0 | 0.384 | 1.966 | Normal-vs-Normal_Weight | **5.78E-03** |
| Extreme weight  (n=88) | 0.007 | 0.35 | 1.244 | Normal-vs-Extreme_Weight | **4.11E-06** |
| Obese  (n=57) | 0.017 | 0.638 | 2.372 | Normal-vs-Obese | **7.04E-02** |
| Extreme obese  (n=11) | 0.026 | 0.887 | 1.74 | Normal-vs-Extreme_Obese | 6.43E-01 |
| **Grade** |  |  |  |  |  |
| Normal  (n=50) | 0.01 | 0.655 | 2.064 | Normal-vs-Grade 1 | 2.60E-01 |
| Grade 1  (n=54) | 0 | 0.459 | 1.966 | Normal-vs-Grade 2 | **2.30E-02** |
| Grade 2  (n=173) | 0 | 0.313 | 1.581 | Normal-vs-Grade 3 | **2.00E-06** |
| Grade 3  (n=118) | 0 | 0.14 | 0.848 | Normal-vs-Grade 4 | **1.58E-03** |
| Grade 4  (n=12) |  |  |  |  |  |
| **Nodal metastasis** |  |  |  |  |  |
| NO  (n=252) | 0 | 0.399 | 1.957 | Normal-vs-N0 | **1.00E-03** |
| N1  (n=4) | 0.366 | 0.385 | 0.527 | Normal-vs-N1 | 5.59E-01 |
| **TP53 mutation** |  |  |  |  |  |
| TP53-Mutant  (n=105) | 0 | 0.362 | 1.611 | Normal-vs-TP53-Mutant | **2.61E-04** |
| TP53-NonMutant  (n=255) | 0.002 | 0.441 | 2.019 | Normal-vs-TP53-NonMutant | **5.30E-03** |
| **Tumor histology** |  |  |  |  |  |
| Hepatocellular carcinoma  (n=361) | 0 | 0.415 | 2.064 | Normal-vs-Hepatocellular carcinoma | **6.00E-04** |
| Fibrolamellar  carcinoma  (n=3) | 0.106 | 0.257 | 1.034 | Normal-vs-Fibrolamellar carcinoma | **6.22E-02** |
| Hepatocholangio carcinoma (Mixed)  (n=7) | 0.746 | 2.019 | 3.575 | Normal-vs-Hepatocholangio carcinoma (Mixed) | 3.03E-01 |

Bold values indicate P < 0.05.

**Supplementary Table 4. Association of FAT4 expression and different clinical factors of HCC patients by Kaplan-Meier plotter**

| Clinicopathological factors | Overall survival | | |  | Progression-free survival | | |
| --- | --- | --- | --- | --- | --- | --- | --- |
|  | N | Hazard ratio | P-value |  | N | Hazard ratio | P-value |
| SEX |  |  |  |  |  |  |  |
| Female | 118 | 0.78 (0.44 -1.39) | 0.4 |  | 120 | **0.51 (0.29 -0.9)** | **0.017** |
| Male | 246 | 0.43 (0.28 -0.67) | **0.00014** |  | 246 | **0.49 (0.34 -0.72)** | **2e-04** |
| AJCC_T |  |  |  |  |  |  |  |
| 1 | 180 | 0.48(0.27 - 0.86) | **0.012** |  | 180 | 0.64 (0.36-1.13) | 0.12 |
| 2 | 90 | 1.31 (0.64 -2.68) | 0.46 |  | 92 | **0.4 (0.23 -0.69)** | **0.00069** |
| 3 | 78 | 0.45(0.24 - 0.85) | **0.011** |  | 78 | **0.56 (0.3 -1.05)** | **0.069** |
| 4 | 13 | - | - |  |  | - | - |
| Vascular invasion |  |  |  |  |  |  |  |
| yes | 90 | 0.58 (0.27 -1.28) | 0.17 |  | 91 | 0.44 (0.25 -0.77) | **0.0036** |
| None | 203 | 0.61(0.36 -1.02) | 0.58 |  | 204 | 1.4 (0.89 -2.2) | 0.14 |
| Race |  |  |  |  |  |  |  |
| White | 181 | 0.7 (0.44 -1.13) | 0.14 |  | 183 | 0.57(0.37 - 0.89) | **0.012** |
| Asian | 155 | 0.26(0.14 - 0.48) | **2.3e-06** |  | 155 | 0.35 (0.22 -0.57) | **8e-06** |
| Alcohol consumption |  |  |  |  |  |  |  |
| yes | 115 | 0.45 (0.22-0.94) | **0.029** |  | 115 | 0.46 (0.27 -0.78) | **0.00034** |
| none | 202 | 0.45(0.28 - 0.71) | **0.00045** |  | 204 | 0.46 (0.3 - 0.71) | **0.00036** |
| Virus hepatitis |  |  |  |  |  |  |  |
| Yes | 150 | 0.26 (0.13 -0.52) | **4.7e-05** |  | 152 | 0.47 (0.29 - 0.77) | **0.02** |
| None | 167 | 0.54(0.34 - 0.85) | **0.0007** |  | 167 | 0.44 (0.28 -0.71) | **0.00043** |

Bold values indicate P < 0.05.

**Supplementary Table 5. Prediction of upstream miRNAs of FAT4 by several target gene prediction programs**

| miRNAname | geneName | clipExpNum | miRmap | microT | miRanda | PicTar | TargetScan |
| --- | --- | --- | --- | --- | --- | --- | --- |
| hsa-miR-17-5p | FAT4 | 1 | 1 | 1 | 1 | 1 | 1 |
| hsa-miR-20a-5p | FAT4 | 1 | 1 | 1 | 1 | 1 | 1 |
| hsa-miR-93-5p | FAT4 | 1 | 1 | 1 | 1 | 1 | 1 |
| hsa-miR-106a-5p | FAT4 | 1 | 1 | 1 | 1 | 1 | 1 |
| hsa-miR-144-3p | FAT4 | 3 | 1 | 1 | 1 | 1 | 1 |
| hsa-miR-193a-3p | FAT4 | 4 | 1 | 1 | 1 | 1 | 1 |
| hsa-miR-106b-5p | FAT4 | 1 | 1 | 1 | 1 | 1 | 1 |
| hsa-miR-20b-5p | FAT4 | 1 | 1 | 1 | 1 | 1 | 1 |

**Supplementary Table 6. Correlation analysis between FAT4 and markers of immune infiltrating cells via GEPIA and TIMER.**

| Description | Gene | HCC | | | | |
| --- | --- | --- | --- | --- | --- | --- |
|  |  | GEPIA | |  | TIMER | |
|  |  | R | P |  | R | P |
| Treg | FOXP3 | 0.15 | **0.0041** |  | 0.343 | ******* |
|  | STAT5B | 0.43 | **0** |  | 0.515 | ******* |
|  | CCR8 | 0.22 | ******* |  | 0.302 | ******* |
| CD4+ T cell | CD27 | 0.014 | 0.79 |  | 0.023 | 0.671 |
|  | CD86 | 0.2 | ******* |  | 0.167 | ******* |
| CD8+ T cell | CD8A | 0.082 | 0.12 |  | 0151 | ******* |
|  | CD8B | 0.0029 | 0.96 |  | -0.005 | 0.925 |
| T cell (general) | CD2 | 0.1 | **0.0045** |  | 0.105 | **0.005** |
|  | CD3D | -0.05 | 0.34 |  | -0.064 | 0.235 |
|  | CD3E | 0.11 | **0.032** |  | 0.134 | **0.001** |
| B cell | CD19 | -0.0033 | 0.95 |  | -0.03 | 0.543 |
|  | CD79A | 0.025 | 0.63 |  | 0.067 | 0.217 |
| Monocyte | CD86 | 0.2 | ******* |  | 0.167 | *** |
|  | CD115 (CSF1R) | 0.26 | ******* |  | 0.215 | *** |
| M1 Macrophage | INOS (NOS2) | 0.12 | **0.02** |  | 0.343 | *** |
|  | IRF5 | 0.19 | ******* |  | 0.185 | *** |
|  | COX2 (PTGS2) | 0.27 | ******* |  | 0.372 | *** |
| M2 Macrophage | CD163 | 0.18 | ******* |  | 0.33 | *** |
|  | VSIG4 | 0.18 | ******* |  | 0.278 | *** |
|  | MS4A4A | 0.25 | ******* |  | 0.264 | *** |
| TAM | CCL2 | 0.15 | 0.003 |  | 0.314 | *** |
|  | CD68 | 0.24 | *** |  | 0.091 | **0.009** |
|  | IL10 | 0.24 | *** |  | 0.107 | **0.005** |
| Natural killer cell | KIR2DL1 | 0.016 | 0.76 |  | 0.039 | 0.471 |
|  | KIR2DL3 | 0.006 | 0.91 |  | 0.044 | 0.413 |
|  | KIR2DL4 | 0.019 | 0.71 |  | -0.014 | 0.799 |
|  | KIR3DL1 | 0.028 | 0.59 |  | 0.182 | ******* |
|  | KIR3DL2 | 0.084 | 0.11 |  | 0.057 | 0.290 |
|  | KIR3DL3 | 0.0056 | 0.92 |  | 0.018 | 0.741 |
|  | KIR2DS4 | 0.0092 | 0.86 |  | 0.049 | 0.364 |
|  | KLRK1 (NKG2D) | 0.089 | 0.089 |  | 0.121 | **0.002** |
|  | NCR1 (NKp46) | 0.12 | 0.021 |  | 0.262 | *** |
|  | NCR2 (NKp44) | 0.025 | 0.63 |  | 0.021 | 0.692 |
|  | NCR3 (NKp30) | 0.077 | 0.14 |  | 0.082 | 0.127 |
| Dendritic cell | HLA-DPB1 | 0.12 | **0.017** |  | 0.154 | ******* |
|  | HLA-DQB1 | -0.017 | 0.74 |  | 0.105 | 0.005 |
|  | HLA-DRA | 0.094 | 0.07 |  | 0.208 | *** |
|  | BDCA1 (CD1C) | 0.32 | *** |  | 0.273 | *** |
|  | BDCA4 (NRP1) | 0.28 | *** |  | 0.395 | *** |
|  | CD11c (ITGAX) | 0.18 | *** |  | 0.155 | *** |
| Neutrophils | CD66b (CEACAM8) | 0.0084 | 0.87 |  | -0.042 | 0.441 |
|  | CD11b(ITGAM) | 0.14 | **0.008** |  | 0.168 | ******* |
|  | CCR7 | 0.09 | 0.084 |  | 0.291 | ******* |
| T-helper 1 cell | T-bet (TBX21) | 0.16 | **0.003** |  | 0.145 | ******* |
|  | STAT4 | 0.22 | ******* |  | 0.152 | ******* |
| T-helper 2 cell | STAT6 | 0.26 | *** |  | 0.321 | ******* |
|  | STAT5A | 0.32 | *** |  | 0.225 | ******* |
|  | IL13 | -0.026 | 0.62 |  | 0.055 | 0.311 |
|  | IL21 | 0.032 | 0.54 |  | -0.008 | 0.878 |
| T-helper 17 cell | IL17A | -0.021 | 0.69 |  | 0.068 | 0.210 |
|  | STAT3 | 0.35 | *** |  | 0.378 | *** |
| T cell exhaustion | PD-1 (PDCD1) | -0.02 | 0.7 |  | 0.378 | *** |
|  | LAG3 | -0.067 | 0.2 |  | -0.029 | 0.595 |
|  | HAVCR2 | 0.19 | *** |  | 0.129 | **0.002** |
| Mast cells | TPSB2 | 0.093 | 0.076 |  | 0.137 | **0.001** |
|  | TPSAB1 | 0.046 | 0.38 |  | 0.132 | **0.001** |
|  | CPA3 | 0.11 | 0.038 |  | 0.231 | *** |
|  | MS4A2 | 0.22 | *** |  | 0.268 | *** |
|  | HDC | 0.16 | 0.0022 |  | 0.154 | *** |

**Supplementary FIgure 1**

**
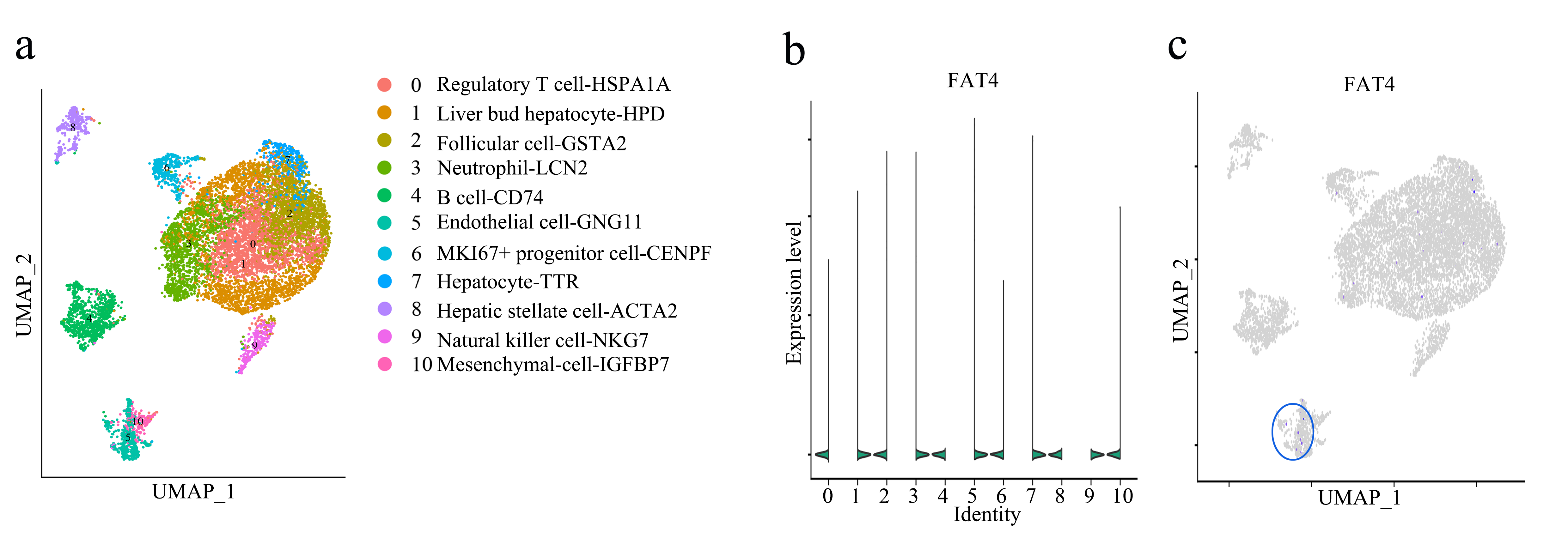
**

**Supplementary FIgure 1:** The single-cell expression pattern and subcellular localization of FAT4 in liver cancer. (a) Cell Distribution Map. (b,c) The FAT4 expression in various clusters is depicted.

**Supplementary FIgure 2**


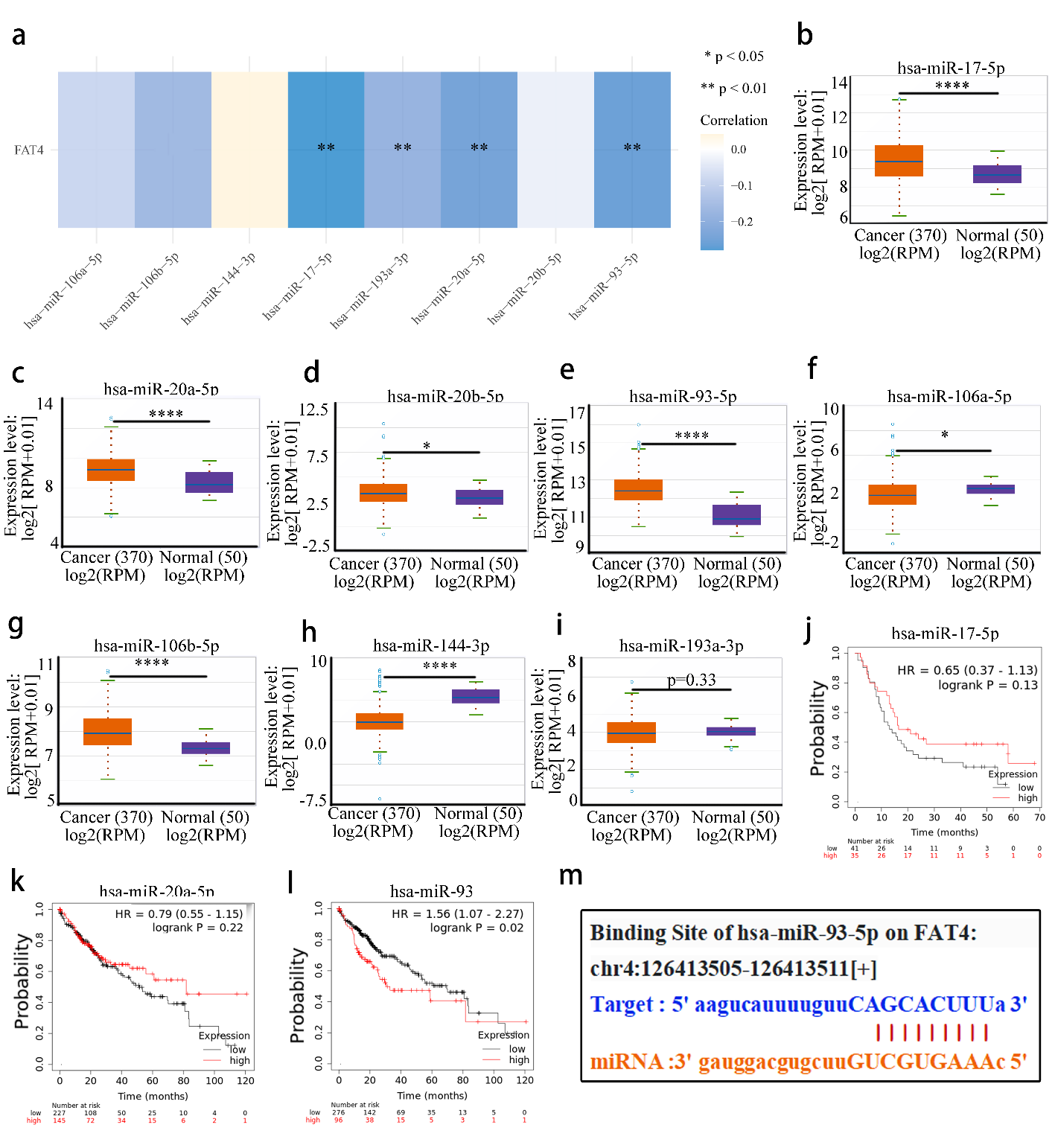


**Supplementary FIgure 2:** FAT4 downregulation correlates with miR-93-5p upregulation in HCC. (a) Correlation of predicted miRNA expression with *FAT4* expression in HCC analyzed using the starBase database. (b–i) Predicted miRNA expression in HCC and normal control samples analyzed using the starBase database. Prognostic significance of (j) hsa-miR-17-5p, (k) hsa-miR-20a-5p, and (l) hsa-miR-93 expression in HCC analyzed using the Kaplan–Meier plotter database. (m) Predicted target region of *FAT4* and hsa-miR-93-5p. *FAT4*, FAT atypical cadherin 4; HCC, hepatocellular carcinoma; HR, hazard ratio; miRNA, microRNA.
